# Demographics and Hospital Outcomes in American Women With Endometriosis and Psychiatric Comorbidities

**DOI:** 10.7759/cureus.9935

**Published:** 2020-08-22

**Authors:** Chris A Robert, Emmanuelle J Caraballo-Rivera, Sasank Isola, Kosisochukwu Oraka, Sabiha Akter, Shikha Verma, Rikinkumar S Patel

**Affiliations:** 1 Obstetrics and Gynecology, Sunrise Hospital, Pune, IND; 2 Medicine, Ross University School of Medicine, Bridgetown, BRB; 3 Medicine, American University of the Caribbean School of Medicine, Cupecoy, SXM; 4 Medicine, Vinnytsia National Medical University, N.I Pirogov, Vinnytsia, UKR; 5 Psychiatry, Bergen New Bridge Medical Center, Paramus, USA; 6 Psychiatry and Behavioral Health, Rosalind Franklin University, North Chicago, USA; 7 Child and Adolescent Psychiatry, Rogers Behavioral Health, Kenosha, USA; 8 Psychiatry, Griffin Memorial Hospital, Norman, USA

**Keywords:** nationwide inpatient sample, sociodemographic differences, depressive disorders, schizophrenia and other psychotic disorders, extended hospitalization stay, psychiatric comorbidities, endometriosis, reproductive age group

## Abstract

Objectives

To explore sociodemographic differences and hospital outcomes in endometriosis patients with versus without psychiatric comorbidities.

Methods

We conducted a cross-sectional study using the Nationwide Inpatient Sample (NIS, 2012-2014), and included 63,160 females with primary diagnosis of endometriosis. We used descriptive statistics and Pearson’s chi-square test to measure the differences in demographics and utilization of gynecologic procedures by the presence of psychiatric comorbidities.

Results

Psychiatric comorbidities were present in 18.7% inpatients with endometriosis. About three-fourth of these inpatients were in reproductive age group 26-45 years (75.7%) and were whites (79.1%). Psychiatric comorbidities were seen more in females from middle-income families and from the midwest region of the US. There was no significant difference in the utilization of gynecological procedures by the presence of psychiatric comorbidities. However, inpatients with psychiatric comorbidities had a longer mean length of stay (2.5 vs. 2.3 days) and total charges ($35,489 vs. $34,673) compared to the non-psychiatric cohort. Anxiety disorders predominated at 45% in patients with endometriosis followed by depressive disorder (31.3%), psychotic disorders (12.3%), and drug abuse (6.3%).

Conclusion

Endometriosis with psychiatric comorbidities is prevalent in young white females from a middle-income family. Anxiety and depressive disorders are most prevalent and are associated with extended hospitalization stay and higher charges, thereby negatively impacting the healthcare burden compared to those without psychiatric comorbidities.

## Introduction

Endometriosis is an estrogen-dependent chronic disorder characterized by the presence of endometrial type glands and stroma outside the uterus [1]. Endometriosis affects an estimated 10 million women in the United States (US), predominantly between the ages of 30 and 40 years [2].

There is an elevated risk of depression and anxiety in women with endometriosis [3-5]. This may be attributed to the consequences of endometriosis, such as chronic pelvic pain, infertility, and subfertility [6]. Endometriosis is often diagnosed later in the disease process, and this may further amplify the psychological suffering in these women and lead to psychiatric disorders [7]. Also, since both endometriosis and psychiatric disorders are heritable, familial risk factors could explain the connection [8].

Since endometriosis is often associated with a spectrum of comorbid conditions, the interactions with other comorbidities could further necessitate additional healthcare expenditure [9-11]. Explaining the role of psychiatric comorbidities, such as depression and anxiety, in the expenditure trajectories of endometriosis patients may aid doctors by raising awareness of the early diagnosis treatment of endometriosis.

In this study, we explore the sociodemographic differences and hospital outcomes in patients with endometriosis with versus without psychiatric comorbidities, and also evaluate the prevalence of spectrum of psychiatric disorders in these inpatients.

## Materials and methods

Data source

We conducted a cross-sectional analysis using the National Inpatient Sample (NIS, 2012 to 2014). The NIS is the largest inpatient data that consists of 4,400 hospitals across 44 states in the US. The primary and comorbid diagnostic information is identified using the International Classification of Diseases, Ninth Revision (ICD-9) and Clinical Classification Software (CCS) codes. As the NIS is a publicly available de-identified data, this study does not require approval from the institutional review board [12].

Inclusion criteria and outcome variables

We included 63,160 inpatients (age 15 to 55 years) with a primary diagnosis of endometriosis using the ICD-9 codes: 617.0-617.9. The study population was further subgrouped based on codiagnosis of psychiatric comorbidities detected in the patient records using codes for attention-deficit/hyperactivity disorder (ADHD, ICD-9 codes: 314.00 or 314.01), psychotic disorders (CCS code: 659), depressive disorders (CCS code: 657), anxiety disorders (CCS code: 651), personality disorders (CCS code: 658), post-traumatic stress disorder (PTSD, ICD-9 code: 309.81), alcohol abuse (CCS code: 660), and drug abuse (CCS code: 661).

We included demographic characteristics (age, race, median household income, and region) and hospital outcomes: length of stay (LOS), i.e. the number of nights the patient was hospitalized for primary diagnosis, total charges (does not include professional fees and non-covered charges), and utilization of gynecologic procedures [13].

Statistical analysis

We used descriptive statistics and Pearson’s chi-square test to measure the differences in demographics and utilization of gynecologic procedures by the presence of psychiatric comorbidities. Independent sample t-test was used for measuring the difference between mean LOS and total charges by the presence of psychiatric comorbidities. A P-value <0.05 was the standpoint for statistical significance in all the analyses that were done on the Statistical Package for the Social Sciences (SPSS) version 26 (IBM Corporation, Armonk, NY).

## Results

We analyzed a total of 63,160 female inpatients managed for endometriosis and 18.74% had psychiatric comorbidities. About three-fourth of these inpatients with psychiatric comorbidities were in the reproductive age group 26 to 45 years (75.7%) and were whites (79.1%). When compared with non-psychiatric comorbidity cohort, psychiatric comorbidities were seen more in females from families with median household income between 26th and 50th percentile (27.2% vs. 25%), and from the midwest region of the US.

There was no significant difference in the utilization of gynecological procedures between both cohorts. However, female inpatients with psychiatric comorbidities had a longer mean LOS (2.5 vs. 2.3 days) and total charges ($35,489 vs. $34,673) compared to the non-psychiatric comorbidity cohort (Table 1).

**Table 1:** Distribution of endometriosis inpatients by psychiatric comorbidities SD: standard deviation

Variable	Psychiatric comorbidities, %		P-value
	(-)	(+)	
Total N	51325	11835	-
Age at admission			
Mean (SD), years	38.7 (7.6)	37.9 (7.7)	<0.001
15–25 years	5.3	5.9	<0.001
26–35 years	27.4	31.4	
36–45 years	47.0	44.3	
46–55 years	29.3	18.4	
Race			
White	63.0	79.1	<0.001
Black	13.3	7.7	
Hispanic	14.1	8.7	
Others	9.5	4.6	
Median household income			
0–25th percentile	25.2	24.1	<0.001
26th–50th percentile	25.0	27.2	
51st – 75th percentile	26.5	26.0	
76th–100th percentile	23.3	22.7	
Region			
Northeast	17.4	18.8	<0.001
Midwest	21.3	25.9	
South	36.8	31.9	
West	24.5	23.4	
Hospital outcomes			
Utilization of gynecologic procedures	4.5	4.3	0.308
Mean length of stay (SD), days	2.3 (1.9)	2.5 (2.4)	<0.001
Mean total charges (SD), $	34673 (28908)	35489 (33058)	0.005

**Figure 1:**
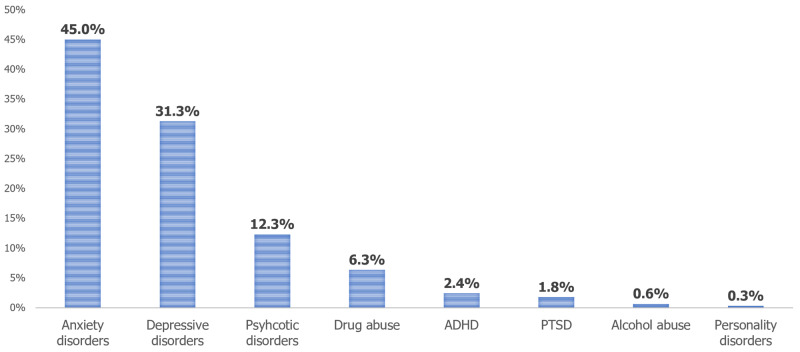
Prevalence of psychiatric comorbidities in endometriosis inpatients ADHD: attention-deficit/hyperactivity disorder; PTSD: post-traumatic stress disorder

Out of the psychiatric comorbidities, anxiety disorders predominated at 45% in patients with endometriosis. This was followed by depressive disorder (31.3%), psychotic disorders (12.3%), and drug abuse (6.3%). Other less prevalent comorbidities included ADHD (2.4%), PTSD (1.8%), alcohol abuse (0.6%), and personality disorder (0.3%) (Figure 1).

## Discussion

Endometriosis has been associated with higher rates of psychiatric comorbidities, such as depression, anxiety, and poor quality of life [14]. About 29% of women with endometriosis present with moderate to severe anxiety, while depression was seen in 14.5% [15]. On the other hand, in a small sample study (N = 104), it was seen that women with pelvic endometriosis had majorly have depressive symptoms (86.5%) and anxiety (87.5%) [4]. In our study, about 18.7% of the inpatients had psychiatric comorbidities, the most common being anxiety disorder (45%) and depressive disorders (31.3%). The reason for this is likely the pain and fertility complications that are symptomatic of endometriosis and chronic pelvic pain [4,16,17]. Moreover, endometriosis being estrogen-dependent, and treatment involves the suppression of estrogen. Therefore, treatment with oral contraceptive pills has led to side effects with decreased psychosexual arousal and adverse changes in mood, and gonadotropin-releasing hormone agonists have been found to be associated with emotional lability and depression [18].

Psychiatric comorbidities were most common in white women of reproductive age between 26 and 45 years and from lower socioeconomic status, which is in line with previous research on women with antepartum mental disorders [19]. Reproductive age in women is a time where the menstrual cycle is actively maximizing the symptoms of endometriosis given the repetitive and consistent nature of the menstrual cycle. Endometriosis has been estimated to affect 10%-15% of women of reproductive age [20]. The relationship between endometriosis and its impact on the patient’s mental health is based on the symptoms of endometriosis, which include dyspareunia, pelvic pain, dysmenorrhea, pain with bowel movements and urination, excessive bleeding, and infertility. These symptoms often affect the psychological and social functioning of patients [4]. In our study, about four-fifth of the endometriosis inpatients were white females, which will require more future studies to identify factors inherent to the white race and related psychosocial factors.

A cross-sectional study of 59,411 women found that the incidence of endometriosis differed by geographic area, being the highest in the south (7%) followed by the midwest (6.4%), the west (5.4%), and the east (5.3%), indicating that a correlation between endometriosis and pigmentary or sun exposure may exist [21]. Our study followed the same trend with the highest incidence of endometriosis with psychiatric comorbidities in the south (31.9%) followed by the midwest (25.9%). This may be attributed to potential correlations between pigment characteristics, melanoma family history, and endometriosis risk [22].

The average annual incremental direct healthcare costs of endometriosis were estimated at $10,002. In contrast, the average incremental indirect costs associated with absence hours and short-term disability compared to non-endometriosis controls were $903 and $1,228, respectively [23]. The incremental costs per patient in the first post-diagnosis year (over $10,000 with multivariable adjustment) were substantial as compared to the estimates for uterine fibroids ($6,873) or menorrhagia ($2,878) in 2014 [24,25]. When associated with a spectrum of comorbid conditions, these could further necessitate additional healthcare expenditure. In our study, we found that there was statistically no significant difference in the utilization of gynecological procedures during hospitalization for endometriosis by the presence of psychiatric comorbidities. However, females with psychiatric comorbidities had significantly longer hospital LOS and higher total charges, which is in line with past inpatient and outpatient studies [19,26]. Past literature also states that psychiatric comorbidities increase the LOS for medical and surgical inpatients, in addition to gynecological inpatients, and increases medical costs leading to more systemic burden on inpatient care [27,28].

There are few limitations of this study being conducted using the administrative database as there is a lack of patient-level clinical information. Yet, the NIS is an excellent population-based representation of diseases related to systematic and temporal factors and has been used in past studies on women's mental health [19,29]. The major strength of this data is the large sample with national representation of the population through a uniform collection of patient records through ICD-9 codes. Since the information is coded independently by the individual practitioners, it was subject to minimal reporting bias.

## Conclusions

Endometriosis with psychiatric comorbidities is prevalent in young white females from a middle-income family. Anxiety and depressive disorders are most prevalent and are associated with extended hospitalization stay and higher charges, thereby negatively impacting the healthcare burden compared to those without psychiatric comorbidities. These comorbidities may worsen the overall symptomatology and disease severity of endometriosis that adversely impacts their relationships, quality of life, and functionality. Early diagnosis and treatment of at-risk patients with psychiatric comorbidities should be done by implementing routine mental health screening for patients with endometriosis to improve their health-related quality of life. Such action from the clinicians and healthcare systems has the potential to improve the mental health of women with endometriosis thereby promoting success in educational, interpersonal, and occupational aspects of life. Future studies should find the prevalence of psychiatric comorbidities in patients diagnosed with endometriosis earlier versus later in life, and the effect that the time of diagnosis has on the severity of illness, hospital outcomes, and the utilization of gynecological procedures.
